# A single extracellular amino acid in Free Fatty Acid Receptor 2 defines antagonist species selectivity and G protein selection bias

**DOI:** 10.1038/s41598-017-14096-3

**Published:** 2017-10-23

**Authors:** Eugenia Sergeev, Anders Højgaard Hansen, Daniele Bolognini, Kouki Kawakami, Takayuki Kishi, Junken Aoki, Trond Ulven, Asuka Inoue, Brian D. Hudson, Graeme Milligan

**Affiliations:** 1 Centre for Translational Pharmacology, Institute of Molecular, Cell and Systems Biology, College of Medical, Veterinary and Life Sciences, University of Glasgow, Glasgow, G12 8QQ Scotland United Kingdom; 2 Department of Physics, Chemistry and Pharmacy, University of Southern Denmark, Campusvej 55, DK-5230 Odense M, Denmark; 3 Graduate School of Pharmaceutical Sciences, Tohoku University, 6–3, Aoba, Aramaki, Aoba-ku, Sendai, 980-8578 Japan; 4 Japan Agency for Medical Research and Development (AMED), Core Research for Evolutional Science and Technology (AMED-CREST), Tokyo, 100-0004 Japan; 5 Japan Science and Technology Agency (JST), Precursory Research for Embryonic Science and Technology (PRESTO), Kawaguchi, Saitama 332-0012 Japan

## Abstract

Free Fatty Acid Receptor 2 is a GPCR activated by short chain fatty acids produced in high levels in the lower gut by microbial fermentation of non-digestible carbohydrates. A major challenge in studying this receptor is that the mouse ortholog does not have significant affinity for antagonists that are able to block the human receptor. Docking of exemplar antagonists from two chemical series to homology models of both human and mouse Free Fatty Acid Receptor 2 suggested that a single lysine - arginine variation at the extracellular face of the receptor might provide the basis for antagonist selectivity and mutational swap studies confirmed this hypothesis. Extending these studies to agonist function indicated that although the lysine - arginine variation between human and mouse orthologs had limited effect on G protein-mediated signal transduction, removal of positive charge from this residue produced a signalling-biased variant of Free Fatty Acid Receptor 2 in which G_i_-mediated signalling by both short chain fatty acids and synthetic agonists was maintained whilst there was marked loss of agonist potency for signalling via G_q/11_ and G_12/13_ G proteins. A single residue at the extracellular face of the receptor thus plays key roles in both agonist and antagonist function.

## Introduction

The role of the microbiota in health and disease is currently attracting enormous interest^1–3^. Among a broad and diverse range of metabolites that the microbiota generate from ingested foodstuffs there has been particular focus on the production of short chain fatty acids (SCFAs) that are generated by fermentation of poorly digested carbohydrates and fiber in the lower gut^4–6^. Whilst SCFAs produced in this manner play wide-ranging roles, including acting as nutrients for colonocytes, the roles that they may play via activating a pair of cell surface G protein-coupled receptors (GPCRs) designated Free Fatty Acid receptor 2 (FFA2) and Free Fatty Acid receptor 3 (FFA3)^7,8^ have attracted particular attention^9–11^. These receptors are expressed by a diverse set of enteroendocrine cells, immune cells, adipocytes and certain peripheral neurons. This expression profile suggests that the receptors might be potential therapeutic targets in disease areas that range from metabolic disorders to inflammatory conditions of the lower gut^8,10,12^.

Previous studies showed that SCFAs produced by the microbiota centred in the colon activate FFA2 expressed in neutrophils and affect mucosal barrier function, resulting in inflammatory conditions of the lower gut, including ulcerative colitis. Thus, FFA2 blockade has been considered as a potential therapeutic target to limit neutrophil infiltration and so alleviate such conditions. Indeed, the FFA2 antagonist 4-[[1-(benzo[*b*]thiophene-3-carbonyl)-2-methylazetidine-2-carbonyl]-(3-chlorobenzyl)amino]butyric acid (GLPG0974) entered phase II clinical trials for treatment of ulcerative colitis but failed to show efficacy^13^. Further development of this ligand was therefore terminated. Nonetheless, both pre-clinical and clinical trials of GLPG0974 were able to show that this compound was able to interact with FFA2 in human volunteers and patients, and effectively blocked chemotaxis of human neutrophils^13,14^. GLPG0974 is the only FFA2 ligand to date that has been employed in clinical studies and its lack of efficacy may indicate that a deeper understanding of the physiological functions of FFA2 is required to develop effective therapeutics. Limiting this desire are observations that GLPG0974 is highly selective for human (h)FFA2 and is unable to effectively block rat or mouse orthologs of this receptor^14,15^. This clearly presents challenges to using GLPG0974 in both rodent-derived cell lines and tissues and in rodent models of disease. The molecular basis for the species ortholog selectivity of GLPG0974 and related molecules has not been defined. Exemplars from a second series of FFA2 antagonists also lack affinity at rodent orthologs of FFA2^16^. A major aim of the current studies was thus to understand the molecular basis for these differences. Using combinations of ligand docking to receptor homology models and direct binding studies using a radiolabelled form of GLPG0974^15^ as well as mutagenesis we show a key role of a single lysine to arginine variation at the extracellular face of human and mouse FFA2 in defining the ability of both antagonist classes to interact with high affinity with human but not mouse FFA2.

In the original studies that identified SCFAs as the endogenous activators of FFA2 the ability of this receptor to signal through multiple classes of heterotrimeric G proteins was highlighted^17^. This is exemplified by the capacity of agonists to promote activation of both G_i_-family G proteins, resulting in reduction in cellular levels of cAMP, and members of the G_q/11_ family, that result in generation of inositol 1, 4, 5 trisphosphate and subsequent elevation of intracellular [Ca^2+^]. The concept of agonist ‘bias’ in promoting one signalling pathway over others has become well established in recent years^18–20^. In the case of FFA2 the agonist *N*-[3-(2-carbamimidamido-4-methyl-1,3-thiazol-5-yl)phenyl]-4-fluoro benzamide (AZ1729) was recently shown to be markedly ‘biased’ in that although an effective and potent regulator of G_i_-mediated signalling it is unable to promote signalling via G_q/11_ family G proteins^21^. Although SCFAs and various synthetic FFA2 agonists can engage with both G protein classes via both human and mouse FFA2^22,23^, removal of positive charge from the same residue position as defined to dictate antagonist binding selection between human and mouse FFA2 produced a form of the receptor in which agonist engagement with both G_q/11_ and G_12/13_ signalling pathways was severely disrupted without hindering those mediated via G_i_ G proteins, generating a highly ‘biased’ form of the receptor. These outcomes illustrate the role of this single extracellular residue in defining both pharmacology and function of this receptor.

## Results

### A single Lysine-Arginine variation defines the human versus mouse selectivity of FFA2 antagonists

**Figure 1:**
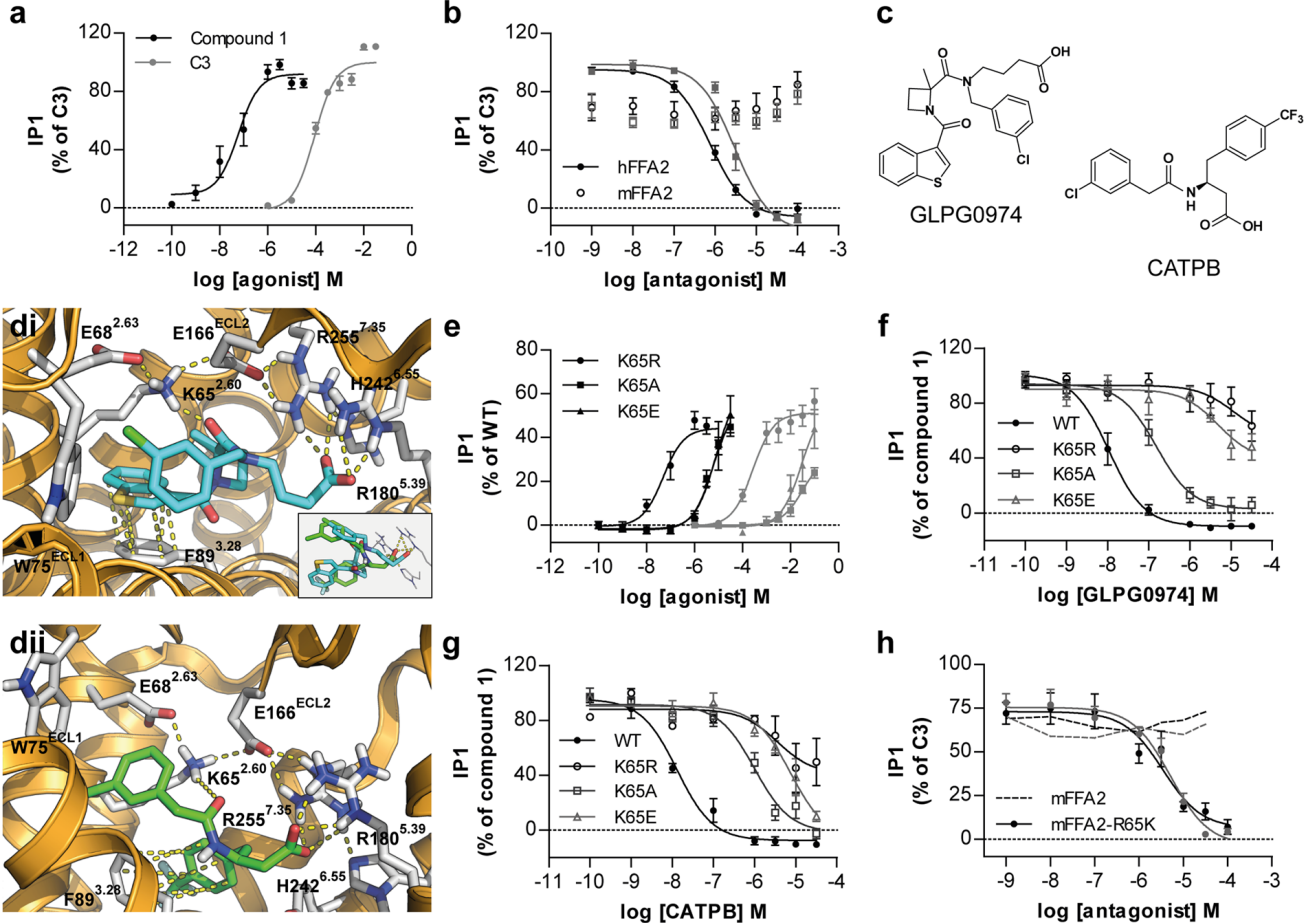
Species selectivity of FFA2 antagonists for human versus mouse ortholog is determined by the identity of residue 65 in FFA2. Receptors of interest were expressed in-frame with eYFP in Flp-In TREx 293 cells. (**a**) The ability of varying concentrations of C3 () and compound 1 (●) to promote production of inositol monophosphates was assessed. (**b**) The capacity of varying concentrations of either GLPG0974 (●) or CATPB () to inhibit inositol monophosphate production induced by EC_80_ concentrations of C3 at either hFFA2 (●) or mFFA2 (○) was assessed. (**c**) Chemical structures of GLPG0974 and CATPB are shown. (**d**) Docking of GLPG0974 (**di**) and CATPB (**dii**) into a homology model of hFFA2 with focus on the potential interaction of each ligand with Lys^65^ (residue 2.60). The insert highlights overlapping poses of GLPG0974 (*cyan*) and CATPB (*green*) within the binding pocket despite their structural differences (**e**). The ability of varying concentrations of C3 () and compound 1 (●) to promote generation of inositol monophosphates in Flp-In TREx 293 cells induced to express Lys^65^Arg (●), Lys^65^Ala (■), and Lys^65^Glu (▲) hFFA2 is illustrated. (**f**,**g**) The ability of GLPG0974 (**f**) or CATPB (**g**) to inhibit inositol monophosphate production induced by EC_80_ concentrations of compound 1 at wild type and each of Lys^65^Arg (○), Lys^65^Ala (□), and Lys^65^Glu (△) hFFA2 is shown. (**h**) The ability of GLPG0974 (●) or CATPB () to inhibit inositol monophosphate production induced by C3 at wild type (*dotted line*) and Arg^65^Lys (●) mFFA2 is illustrated.

Following doxycyline-induced expression of hFFA2 in Flp-In TREx 293 cells the endogenous SCFA propionate (C3) and the synthetic FFA2 agonist 3-benzyl-4-(cyclopropyl-(4-(2,5-dichlorophenyl)thiazol-2-yl)amino)-4-oxobutanoic acid (compound 1)^23^ were both able to promote accumulation of inositol monophosphates (Fig. 1a), with compound 1 being in the region of 1000 times more potent than C3 (Table 1). This effect was prevented by increasing concentrations of either of two exemplar hFFA2 antagonists, GLPG0974^14^ and (*S*)-3-(2-(3-chlorophenyl)acetamido)-4-(4-(trifluoromethyl)phenyl)butanoic acid (CATPB)^22^ (Fig. 1b), that derive from distinct chemical series (Fig. 1c)^8^. By contrast, when equivalent experiments were performed following expression of the mouse ortholog of FFA2 (mFFA2) C3**-**stimulated inositol monophosphate production was unaffected by either GLPG0974 or CATPB (Fig. 1b). Although structurally quite distinct (Fig. 1c), ligand docking studies using a homology model of hFFA2 previously indicated that the carboxylate moiety of each of these compounds interact, if differentially^15^, with a pair of arginines (residue numbers 180 and 255, positions 5.39 and 7.35, respectively in the Ballesteros and Weinstein^24^ residue location system) that also interact with the carboxylate of both C3 and compound 1. Further analysis of the ligand docking studies also indicated the potential for a charge-assisted hydrogen bond between one of the two amide-carbonyls of GLPG0974 and Lys^65^ of the receptor (Fig. 1di). Similarly, this also predicted Lys^65^ to participate in a charge-assisted hydrogen bond with the single amide-carbonyl of CATPB (Fig. 1dii). Overall, despite the structural differences between CATPB and GLPG0974 these interactions resulted in overlapping poses of the two antagonists (Fig. 1d **insert**).

**Table 1:** Lys^65^Xaa alterations in hFFA2 affect both agonist potency to generate inositol monophosphates and the ability of antagonists to inhibit this response.

Compound	WT	K65R	K65A	K65E
C3	4.06 ± 0.08	3.71 ± 0.05^*^	<2.5	<2.5
Compound 1	6.98 ± 0.06	6.70 ± 0.07^*^	<5.5	<5.5
GLPG0974	7.92 ± 0.19	<4.5	7.36 ± 0.17	<4.5
CATPB	7.72 ± 0.14	<4.5	6.36 ± 0.19	<4.5

Values represent pEC_50_ for agonists C3 and compound 1 and pIC_50_ values for antagonists GLPG0974 and CATPB against an EC_80_ concentration of compound 1. Data are mean ± SEM with n ≥ 3. ^*^p ≤ 0.05. One-way analysis of variance was followed by Dunnett’s test with WT as reference.

Lys^65^ is located at the extracellular surface, at the very top of transmembrane domain II (position 2.60 in the nomenclature of Ballesteros and Weinstein^24^) of hFFA2. Although Lys^65^ lies outwith the previously identified core orthosteric agonist binding pocket of hFFA2^25^ mutation of this residue to either Ala or to Glu resulted in a substantial loss of potency for both C3 and compound 1 in inositol monophosphate accumulation assays (Fig. 1e, Table 1). The modest potency of C3 at wild type hFFA2 and the limited solubility of compound 1 made assessment of the true extent of loss of potency at the Ala and Glu substitutions challenging to quantify accurately (Table 1) but in each case it was greater than 30 fold. By contrast, following mutation of Lys^65^ to Arg in hFFA2 only a small, but still statistically significant, reduction in potency of both C3 and compound 1 was observed (Fig. 1e, Table 1). Although the potency of the two tested agonist compounds was reduced substantially at both the Lys^65^Ala and Lys^65^Glu mutants of hFFA2, each mutant still responded at sufficiently high concentrations of compound 1 to allow assessment of the potential ability of GLPG0974 (Fig. 1f) and CATPB (Fig. 1g) to act as antagonists at these mutants. Both antagonists lost substantial potency at each of these two point mutants of hFFA2 (Table 1). Moreover, at Lys^65^Arg hFFA2 the ability of both GLPG0974 and CATPB to block compound 1**-**mediated elevation of inositol monophosphate accumulation was all but abolished (Fig. 1f,g). This was of particular interest because in mFFA2 residue^65^ is Arg rather than Lys. Docking of either GLPG0974 or CAPTPB to an equivalent homology model of mFFA2 resulted in poses for each ligand that were devoid of interactions with Arg^65^ (**not shown**) which, rather, was fixed in an ionic-lock conformation with residue Glu^68^ (Ballesteros and Weinstein residue position 2.63). In concert with the functional data this was potentially consistent with the Lys^65^-Arg^65^ variation between hFFA2 and mFFA2 being responsible for the lack of function of both GLPG0974 and CATPB as antagonists at mFFA2 (Fig. 1b). To test this directly we generated the reverse mutation in which Arg^65^ in mFFA2 was converted to Lys. Gratifyingly, following expression of Arg^65^Lys mFFA2, both GLPG0974 and CATPB were able to fully antagonize C3-mediated inositol monophosphate accumulation, in a concentration-dependent manner (Fig. 1h).

**Figure 2:**
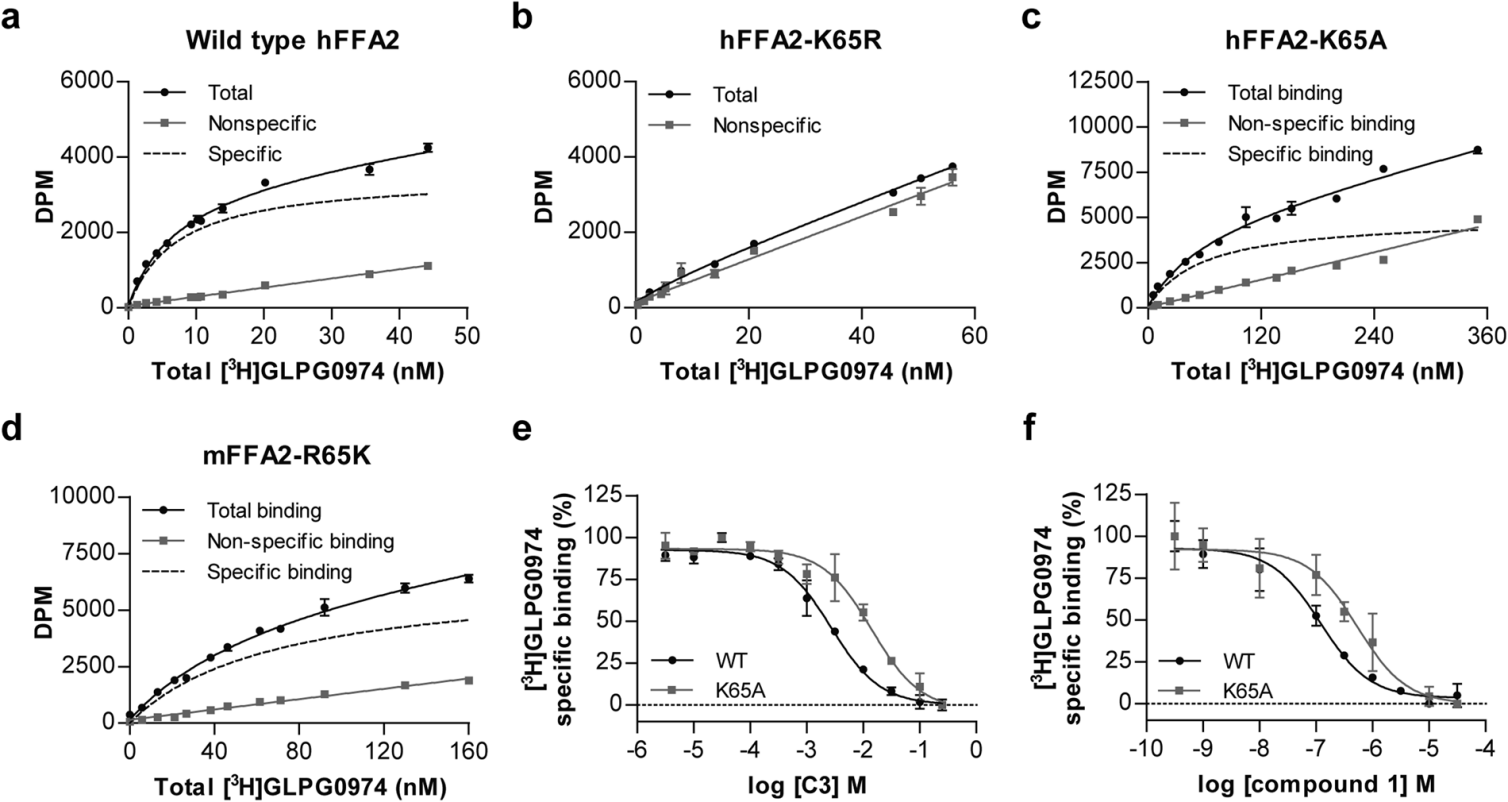
Binding of [^3^H]GLPG0974 to hFFA2 and various mutants. Total, nonspecific and specific (total minus nonspecific) binding of the indicated concentrations of [^3^H]GLPG0974 to membranes of Flp-In TREx 293 cells induced to express wild type hFFA2 (**a**), Lys^65^Arg hFFA2 (**b**), Lys^65^Ala hFFA2 (**c**) or Arg^65^Lys mFFA2 (**d**) is shown from representative experiments. The ability of C3 (**e**) and compound 1 (**f**) to compete with approximate K_d_ concentrations of [^3^H]GLPG0974 to bind to membranes expressing wild type (●) or Lys^65^Ala () hFFA2 is illustrated.

To extend these observations we performed sets of specific binding studies using the radioligand [^3^H]GLPG0974^15^. [^3^H]GLPG0974 bound with high affinity (K_d_ = 7.3 ± 0.4 nM) to hFFA2 (Fig. 2a). By contrast, no specific binding to Lys^65^Arg hFFA2 could be detected at up to 60 nM [^3^H]GLPG0974 (Fig. 2b). Moreover, in line with the reduced potency of GLPG0974 to inhibit agonist-induced inositol monophosphate accumulation at Lys^65^Ala hFFA2 (Fig. 1f, Table 1), direct binding studies using [^3^H]GLPG0974 indicated that affinity of the radioligand at this mutant was reduced some 10 fold (K_d_ = 67 ± 9 nM) compared to wild type hFFA2 (Fig. 2c). Consistent with the functional inhibition produced by GLPG0974 at Arg^65^Lys mFFA2, direct binding studies with [^3^H]GLPG0974 showed the radioligand to bind with high affinity to this mutant of the mouse ortholog of FFA2 (Fig. 2d). However, as the measured affinity (K_d_ = 51 ± 11 nM) was some 7 fold lower than for wild type hFFA2, this single variation in sequence was unable to account fully for the human versus mouse FFA2 species differences in responsiveness to GLPG0974. Although loss of potency of both C3 and compound 1 was extensive at Lys^65^Ala hFFA2 when measured in inositol monophosphate accumulation assays (Table 1) the ability of each of these two agonists to bind to the Lys^65^Ala hFFA2 variant, as assessed by their ability to compete for binding with [^3^H]GLPG0974, was much less affected (Fig. 2e,f).

### Removal of positive charge from residue 65 of Free Fatty Acid receptor 2 skews agonist-induced G protein selection

**Figure 3:**
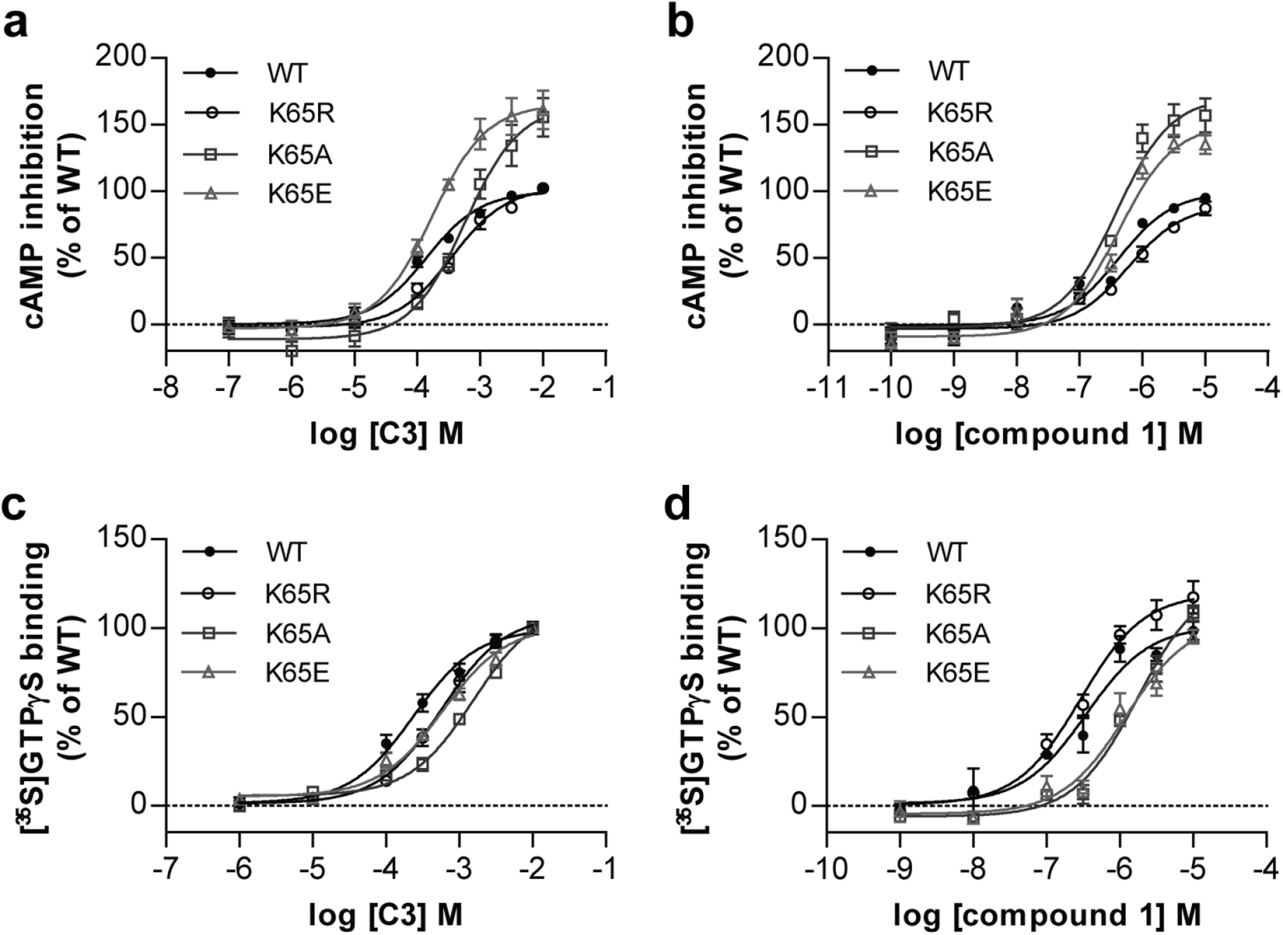
Agonist responses in assays that reflect activation of G_i_-G proteins are only modestly affected by charge-modifying mutations of Lys^65^ in hFFA2. The ability of C3 (**a**,**c**) and compound 1 (**b**,**d**) to promote inhibition of forskolin-amplified cAMP levels (**a**,**b**) or to enhance binding of [^35^S]GTPγS (**c**,**d**) at each of wild type hFFA2 (●), Lys^65^Arg hFFA2 (○), Lys^65^Ala hFFA2 (□) or Lys^65^Glu (△) hFFA2 is illustrated.

We next assessed the implications and potential basis of the apparent dichotomy between measured agonist binding affinity and functional potency in inositol monophosphate accumulation studies. Inositol monophosphate accumulation produced by agonists of FFA2 following expression of the receptor in HEK293 cells reflects activation of G_q/11_ heterotrimeric G proteins^26^. However, it is well established that FFA2 can also interact functionally with other heterotrimeric G proteins, including members of the pertussis toxin sensitive G_i_-family^17,21^. We, therefore, also assessed agonist function at each of wild type hFFA2 and the set of Lys^65^-Xaa hFFA2 mutants described above in two distinct assays that each reflects activation of G_i_-family G proteins. When measuring the ability of C3 to inhibit forskolin-amplified cAMP levels in intact cells expressing each variant the potency of the SCFA was unaffected (Lys^65^Glu), or only modestly reduced (Lys^65^Ala, Lys^65^Arg), compared to the wild type receptor (Fig. 3a, Table 2). For compound 1 this was even more marked with no significant reduction in potency noted at any of Lys^65^Glu, Lys^65^Arg or Lys^65^Ala hFFA2 (Fig. 3b, Table 2). However, for both the Lys^65^Glu and Lys^65^Ala hFFA2 mutants the maximal effect of both C3 and compound 1 was greater than at wild type hFFA2 (Fig. 3a,b). In [^35^S]GTPγS binding assays performed on membrane preparations of cells expressing these variants of hFFA2 this broad pattern was retained, with effects on potency of both C3 and compound 1 limited to less than 6-fold (Fig. 3c and d, Table 2).

**Table 2:** Lys^65^Xaa alterations in hFFA2 have limited effects in assays that are transduced via G_i_-G proteins.

Assay	Agonist	WT	K65R	K65A	K65E
cAMP	C3	3.90 ± 0.09	3.42 ± 0.06^**^	3.20 ± 0.11^***^	3.79 ± 0.08
	Compound 1	6.37 ± 0.06	6.18 ± 0.08	6.54 ± 0.10	6.47 ± 0.13
	AZ1729	6.35 ± 0.13	6.47 ± 0.15	6.42 ± 0.03	6.45 ± 0.15
GTPγS	C3	3.59 ± 0.08	3.29 ± 0.08^*^	2.83 ± 0.06^***^	3.21 ± 0.05^**^
	Compound 1	6.46 ± 0.04	6.52 ± 0.06	5.68 ± 0.08^***^	5.98 ± 0.07^***^
	AZ1729	6.97 ± 0.02	6.79 ± 0.07	6.94 ± 0.14	6.79 ± 0.12
Expression (% of WT)		100 ± 1	187 ± 3	96 ± 1	97 ± 1

Values represent pEC_50_ of agonists in respective assays. Data are mean ± SEM with n ≥ 3. ^*^p ≤ 0.05. ^**^p ≤ 0.01. ^***^p ≤ 0.001. One-way analysis of variance was followed by Dunnett’s test with WT as reference.

**Figure 4:**
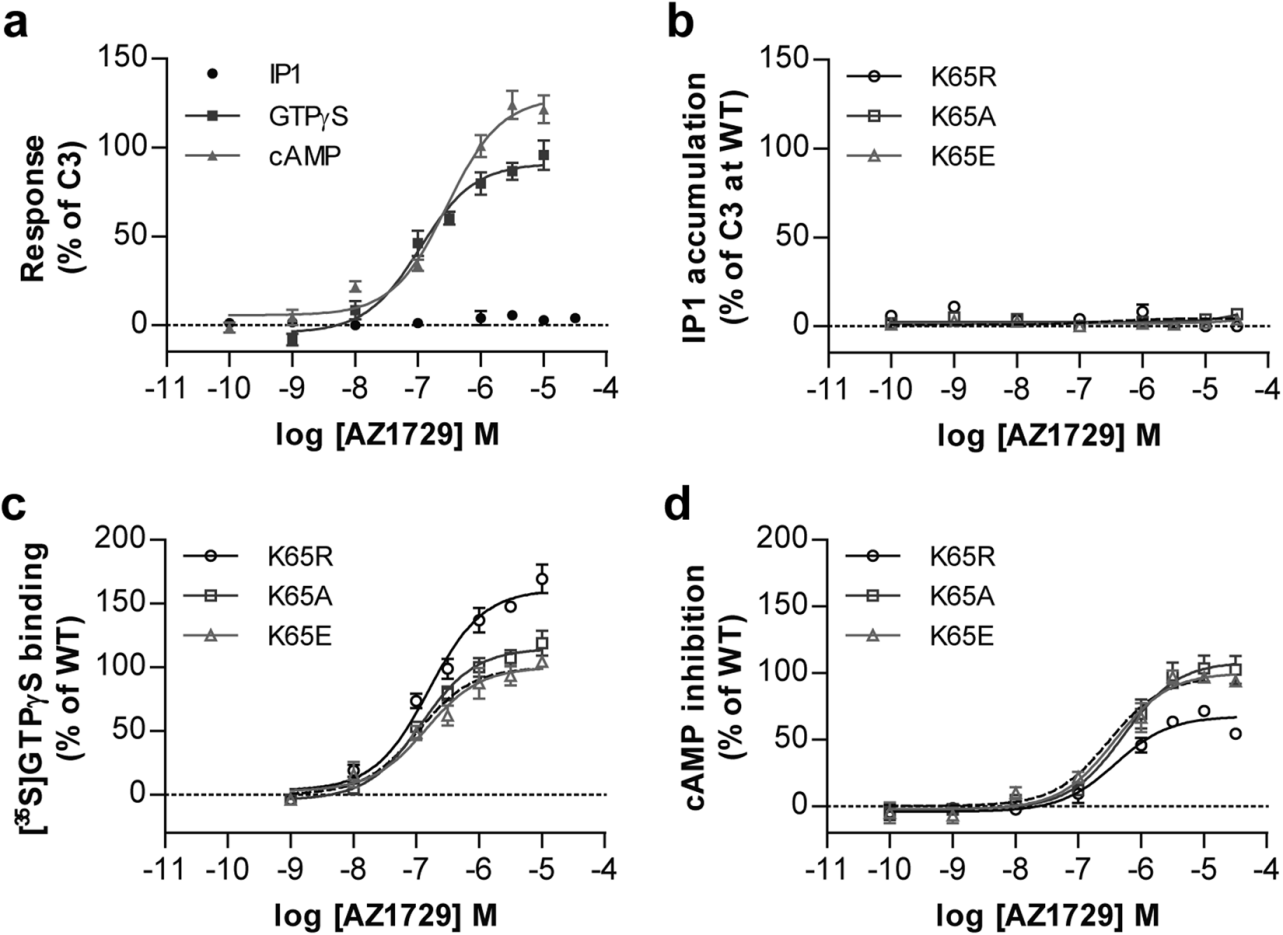
Signalling of AZ1729 is unaffected by mutation of Lys^65^ in hFFA2. The ability of varying concentrations of AZ1729 to regulated cAMP levels (), promote binding of [^35^S]GTPγS () and promote production of inositol monophosphates (●) in cells or membranes induced to express hFFA2 is illustrated (**a)**. (**b**) AZ1729 is unable to promote inositol monophosphate production via Lys^65^Arg hFFA2 (○), Lys^65^Ala hFFA2 (□) or Lys^65^Glu (△) hFFA2. (**c**,**d**) Potency of AZ1729 is unaffected at Lys^65^Arg hFFA2 (○), Lys^65^Ala hFFA2 (□) or Lys^65^Glu (△) hFFA2 in [^35^S]GTPγS (**c**) or cAMP inhibition (**d**) studies.

These modest changes in agonist potency in measures of G_i_-mediated function compared to those observed in G_q/11_-mediated inositol monophosphate accumulation studies suggested that mutation of Lys^65^ in hFFA2 might generate a ‘biased’ form of the receptor, in which effects mediated via G_q_/G_11_ were altered more extensively than effects transduced via G_i_-family G proteins. To test this hypothesis directly we first employed the FFA2 agonist AZ1729. We have previously shown that this ligand is able to transduce signals only via G_i_-mediated pathways and not via G_q_/G_11_ pathways^21^. As anticipated from this AZ1729 was unable to promote accumulation of inositol monophosphates via hFFA2 (Fig. 4a). By contrast AZ1729 both increased binding of [^35^S]GTPγS (Fig. 4a, Table 2) and inhibited forskolin-stimulated levels of cAMP (Fig. 4a, Table 2) in a concentration-dependent manner. Alteration of Lys^65^ to Ala, Arg or Glu did not promote an ability of AZ1729 to stimulate inositol monophosphate accumulation (Fig. 4b) whilst, notably, the potency of AZ1729 in both of the G_i_-coupled assays was completely unaffected by alteration of Lys^65^ to Ala, Arg or Glu (Fig. 4c and d, Table 2). This indicates that coupling of hFFA2 to G_i_-mediated end points is intrinsically unaffected by mutation of residue Lys^65^.

**Figure 5:**
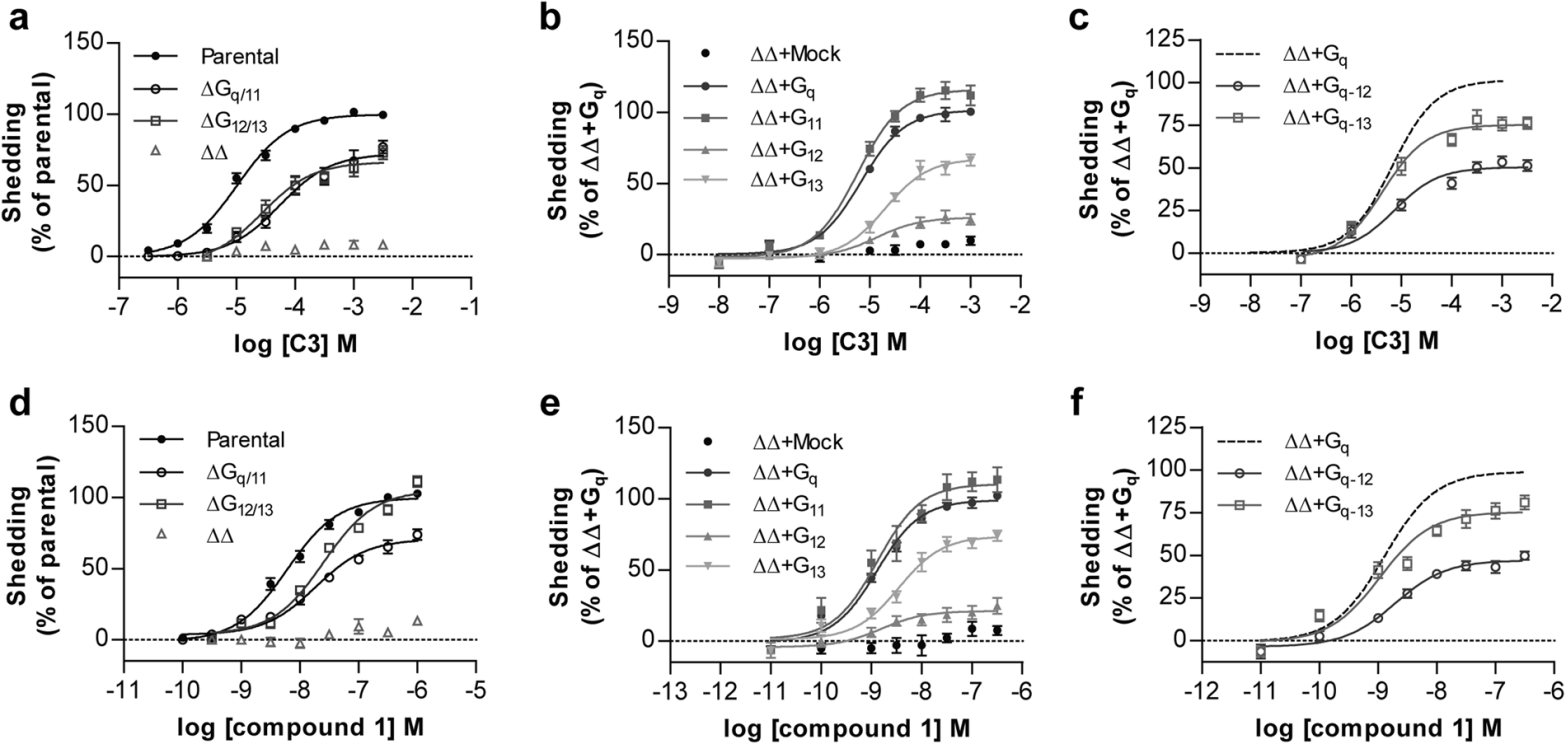
Activation of hFFA2 by C3 and by compound 1 induces TGFα shedding via both G_q/11_ and G_12/13_ (a,d) Varying concentrations of C3 (**a**) or compound 1 (**d**) were able to promote shedding of TGFα via hFFA2 in each parental HEK293 cells and those genome edited to lack (Δ) expression of G_q_+G_11_, or G_12_+G_13_ but not in cells genome edited to lack all four of these G protein α subunits (ΔΔ). (**b**,**e**) Reintroduction of each of Gα_q_, Gα_11_, Gα_12_ and Gα_13_ into cells lacking all four of these G proteins allowed reconstitution of TGFα shedding via FFA2 by both C3 (**b**) and compound 1 (**e**). (**c**,**f**) Introduction of chimeric Gα_q−12_ or Gα_q−13_ into HEK293 cells genome edited to lack each of Gα_q_, Gα_11_, Gα_12_ and Gα_13_ resulted in effective shedding of TGFα in response to C3 (**c**) and compound 1 (**f**).

To examine potential shifts in G protein selection produced by mutation of Lys^65^ in hFFA2 more fully we next employed a transforming growth factor-α (TGFα) shedding assay^27^. Following transfection of wild type hFFA2 into parental HEK293 cells alongside a membrane-bound pro-form of alkaline phosphatase (AP)-tagged TGFα, addition of C3 resulted in shedding of AP-TGFα into the cell medium (Fig. 5a). This occurred in a concentration-dependent manner with pEC_50_ = 4.93 ± 0.04 (Table 3), notably some 10 fold higher than in the inositol monophosphate accumulation assay. The TGFα shedding assay integrates information on signal transduction mediated via both G_q/11_ and G_12/13_ G proteins^27^ and previous studies, in assays using *Saccharomyces cerevisiae*-based chimeric G proteins, have indicated that FFA2 can activate G_12/13_ as well as G_q/11_ G proteins^17^. We, therefore, used the TGFα shedding assay system to investigate hFFA2 coupling to endogenous G_12/13_ G proteins and also to assess potential effects of mutation of Lys^65^ in hFFA2 on coupling of the receptor to members of this G protein subfamily. To resolve outcomes in the TGFα shedding assay that reflect coupling to G_q/11_ from those that reflect coupling to G_12/13_ the TGFα shedding assays were performed in HEK293 cells genetically modified by CRISPR/Cas9 genome editing. These cell lines either lacked expression of both Gα_q_ and Gα_11_^26,28^, and therefore signals are limited to those generated via G_12_ and/or G_13_, or lacked expression of both G_12_ and G_13_^29^ and, therefore, generate signals only via G_q_ and/or G_11_. In each of these genome-edited cell lines shedding of TGFα was also promoted by C3 in a concentration-dependent manner (Fig. 5a), although in both these cases the potency of C3 was lower than in equivalent assays performed in parental HEK293 cells (Table 3). Consistent with the concept that the TGFα shedding assay reflects only activation of these specific G protein subgroups, when similar assays were performed in HEK293 cells in which expression of all four of these G protein α subunits (Gα_q_, Gα_11_, Gα_12_, Gα_13_) had been eliminated^30^, no TGFα shedding response to C3 was produced (Fig. 5a). To assess the signalling profile of hFFA2 in further detail, the HEK293 cells genome-edited to lack all four of the Gα subtypes that can induce the TGFα shedding response were employed and transfected individually with each of Gα_q_, Gα_11_, Gα_12_ and Gα_13_, alongside wild type hFFA2. Each of the four specific G protein α subunits (Gα_q_, Gα_11_, Gα_12_, Gα_13_) was able to reconstitute C3-mediated function (Fig. 5b). However, the signal produced in the presence of Gα_q_ or Gα_11_ was substantially greater than for Gα_13_ and, particularly, for Gα_12_ (Fig. 5b). It was uncertain from these data if the poor response in the presence of Gα_12_ and, to a lesser extent, Gα_13_ reflected weak coupling of these G proteins to hFFA2 compared to Gα_q_ and Gα_11_ or poor downstream coupling to the mechanisms of induced TGFα shedding. To define this we employed chimeric G proteins consisting of the backbone of Gα_q_ with substitution of the C-terminal six amino acids of Gα_q_ with the corresponding sequence from Gα_12_ or Gα_13_ because this region of the G protein C-terminal α5 helix defines receptor-G protein selection^31^. Now, introduction of AP-tagged TGFα and hFFA2 alongside Gα_q−13_ and Gα_q−12_ chimeric G proteins resulted in substantially more robust shedding of TGFα induced by C3 than observed when using full length Gα_12_ or Gα_13_ (Fig. 5c). This suggests that Gα_12_ in particular interacts poorly with the mechanisms that induce TGFα shedding but this G protein does couple effectively to hFFA2. Equivalent outcomes were produced when using compound 1 as agonist rather than C3 in each of these assay formats (Fig. 5d–f). As for C3, the potency of compound 1 in the TGFα shedding assay performed in parental HEK293 cells was also some 10 fold higher than in the inositol monophosphate studies (compare Table 3 and Table 1).

**Table 3:** Potency of FFA2 agonists in the TGFα shedding assay and effects of Lys^65^Xaa mutations.

Cells	Agonist	WT	K65R	K65A	K65E
Parental	C3	4.93 ± 0.04	4.68 ± 0.09	2.23 ± 0.13^***^	2.79 ± 0.07^***^
	Compound 1	8.12 ± 0.08	8.14 ± 0.03	6.88 ± 0.04^***^	6.88 ± 0.04^***^
ΔG_q/11_	C3	4.40 ± 0.07^aaa^	3.98 ± 0.04^*^	2.67 ± 0.17^***^	2.39 ± 0.07^***^
	Compound 1	7.84 ± 0.08^a^	7.51 ± 0.10	6.02 ± 0.11^***^	5.94 ± 0.08^***^
ΔG_12/13_	C3	4.38 ± 0.10^aaa^	4.11 ± 0.09	2.14 ± 0.08^***^	2.52 ± 0.05^***^
	Compound 1	7.49 ± 0.08^aaa^	7.33 ± 0.11	6.23 ± 0.11^***^	6.16 ± 0.13^***^

Values represent pEC_50_ of agonists in denoted cell lines. Data are mean ± SEM with n ≥ 3. ^*/a^p ≤ 0.05. ^**/aa^p ≤ 0.01. ^***/aaa^p ≤ 0.001. One-way analysis of variance was followed by Dunnett’s test with WT (^*^) or parental cells (^a^) as reference.

**Figure 6:**
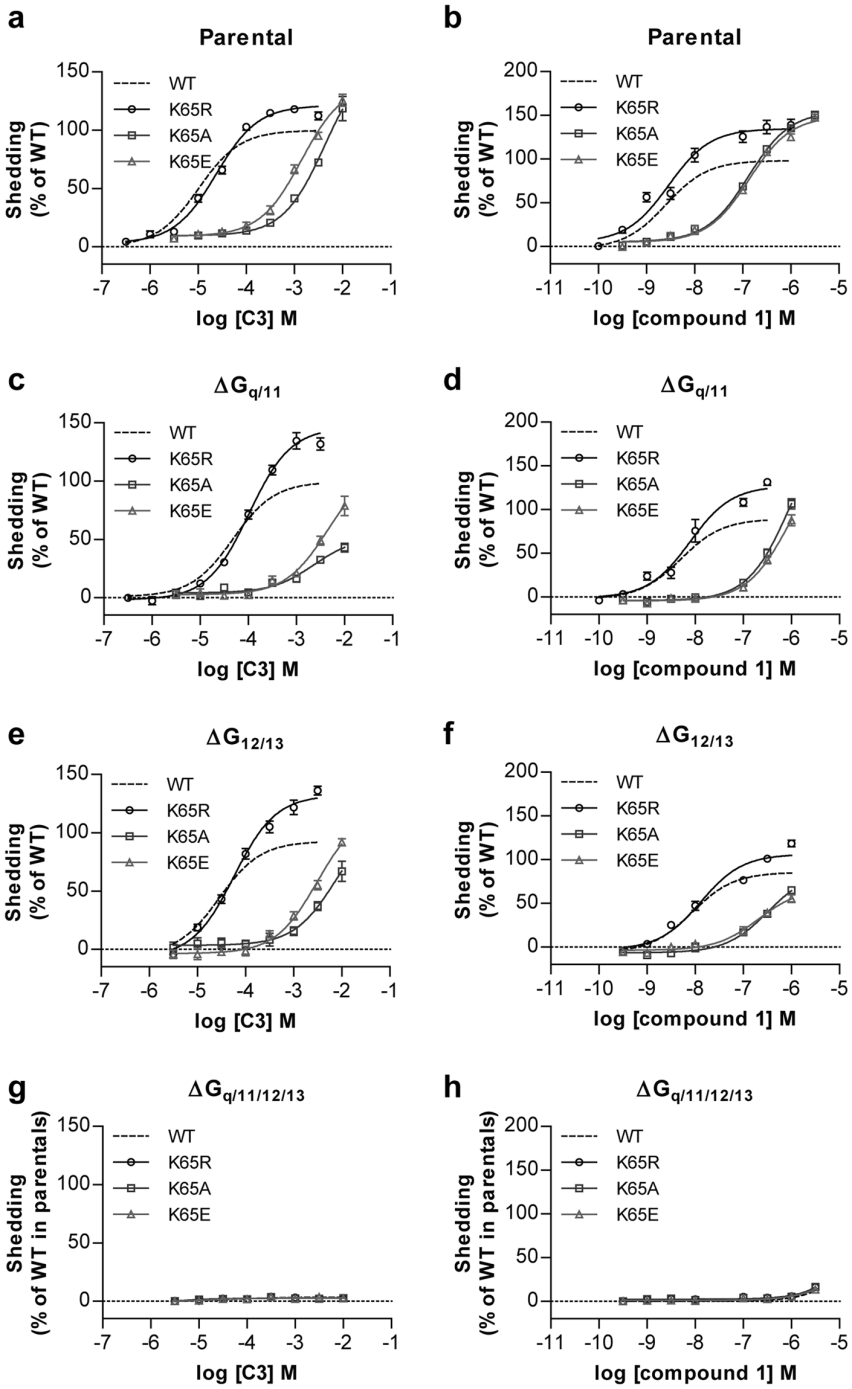
Removal of positive charge from residue^65^ in hFFA2 affects response to agonists in G_q/11_- and G_12/13_-coupled TGFα shedding assays in a manner akin to inositol monophosphate assays. TGFα shedding assays were performed in response to varying concentrations of C3 (**a**,**c**,**e**,**g**) or compound 1 (**b**,**d**,**f**,**h**) in parental HEK293 cells (**a**,**b**), or those genome-edited to lack both Gα_q_ and Gα_11_ (**c**,**d**), both Gα_12_ and Gα_13_, (**e**,**f**) or each of Gα_q_, Gα_11_, Gα_12_ and Gα_13_ (**g**,**h**) and transfected to express either wild type hFFA2 (*dotted lines*) or Lys^65^Arg hFFA2 (○), Lys^65^Ala hFFA2 (□) or Lys^65^Glu (△) hFFA2.

Given that the genome-edited HEK293 cell lines allowed separate assessment of coupling of hFFA2 to the G_q/11_ and G_12/13_ G protein subfamilies, we next assessed whether mutation of Lys^65^ in hFFA2 also differentially ‘biased’ coupling between these G protein subsets. It did not: In each of G_12/13_-deleted and G_q/11_-deleted HEK293 cell lines Lys^65^Arg hFFA2 had no, or very limited, effect on the potency and function of either C3 or compound 1 (Fig. 6, Table 3). By contrast, for both Lys^65^Ala hFFA2 and Lys^65^Glu hFFA2 large, and similar, reductions in potency for both agonists was observed (Fig. 6, Table 3).

Usefully, the noted higher potency of both agonist ligands at wild type hFFA2 in this assay compared to the inositol monophosphate studies resulted in improved data quality and measurement of the extent of loss of agonist potency at Lys^65^Ala hFFA2 and Lys^65^Glu hFFA2 (Fig. 6, Table 3). This allowed efficient calculation of the degree of ‘bias’ between G_i_ and G_q_/G_11_ and G_12_/G_13_ signalling imbued by alteration of Lys^65^ to either Ala or Glu in hFFA2 (Table 4). These showed that for the endogenously generated SCFA C3, alteration of Lys^65^ to either Ala or Glu resulted in receptor function that was biased between 41–60 fold to favor G_i_-mediated signalling compared to wild type hFFA2 when calculations were based on the data from [^35^S]GTPγS binding studies, and between 100–125 fold when using data from cAMP inhibition studies (Table 4). Equivalent calculations based on the function of compound 1 resulted in assessed bias towards G_i_-mediated pathways being less extensive, but still between 21–27 fold when using values derived from cAMP inhibition studies (Table 4).

**Table 4:** Agonist ‘bias’ factors for Lys^65^Xaa alterations in hFFA2.

Pathway 1	Pathway 2	Agonist	β factor (compared to WT)		
			K65R	K65A	K65E
cAMP	TGFα shedding	C3	−0.30	2.00	2.10
		Compound 1	−0.39	1.44	1.33
GTPγS	TGFα shedding	C3	−0.10	1.78	1.62
		Compound 1	−0.03	0.36	0.59

## Discussion

Although in general not well characterized, there are a number of examples in which synthetic, small molecule, pharmacological tool compounds display marked variation in interactions with species orthologs of members of the G protein-coupled receptor superfamily. As initial screens for function invariably use the human receptor ortholog expressed in heterologous cell systems, this can create major challenges for understanding the basic underpinning biological roles of a receptor in animal models, as such efforts frequently centre on rodents and tissue derived from this group of animals. An example is that all currently described antagonists of FFA2, whilst displaying moderately high affinity at the human ortholog are not able to effectively block mouse or rat FFA2^9,16^. Although atomic level structures of FFA2 are not available, structures of the related free fatty acid receptor 1 (FFA1) have been published^32,33^. We, therefore, initially developed homology models of both human and mouse FFA2 and attempted to dock the most widely studied exemplars, GLPG0974 and CATPB, of the two currently available antagonist series^16^ into each. These studies confirmed previously identified interactions of the carboxylate group present in both antagonist molecules with the pair of arginine residues located at positions 5.39 and 7.35 in hFFA2, as well as the relative preference for interaction of GLPG0974 with arginine 5.39 and of CATPB with arginine 7.35^15^. However, replacement of the carboxylate of both CATPB, and of an antagonist closely related to GLPG0974 with a methyl ester has been shown to result in only relatively modest reductions in ligand affinity^15^. This implies important roles for other residues in hFFA2 in recognition and binding affinity of CATPB and GLPG0974. For both, further interactions were highlighted herein in ligand docking studies using the homology model of hFFA2. The benzothiophene moiety of GLPG0974, a group that is critical for binding of GLPG0974-type compounds containing 3-chlorophenyl^14^, was sandwiched between Phe^89^ (residue location 3.28) (π-stacking interaction) and Lys^65^ (residue location 2.60) (π–cation interaction) (Fig. 1di). Lys^65^ also was able to form a charge-assisted hydrogen bond to one of the amide carbonyls of GLPG0974 (Fig. 1di), locking the compound in place within the binding site. For CATPB, Lys^65^ of hFFA2 was also able to form a charge-assisted hydrogen bond to the single amide carbonyl (Fig. 1dii). Moreover, this residue also generated a π–cation interaction with the 3-chlorophenyl group of the antagonist (Fig. 1dii). Furthermore, the trifluoromethylphenyl of CATPB engaged Phe^89^ through π-stacking interactions (Fig. 1dii).

**Figure 7:**
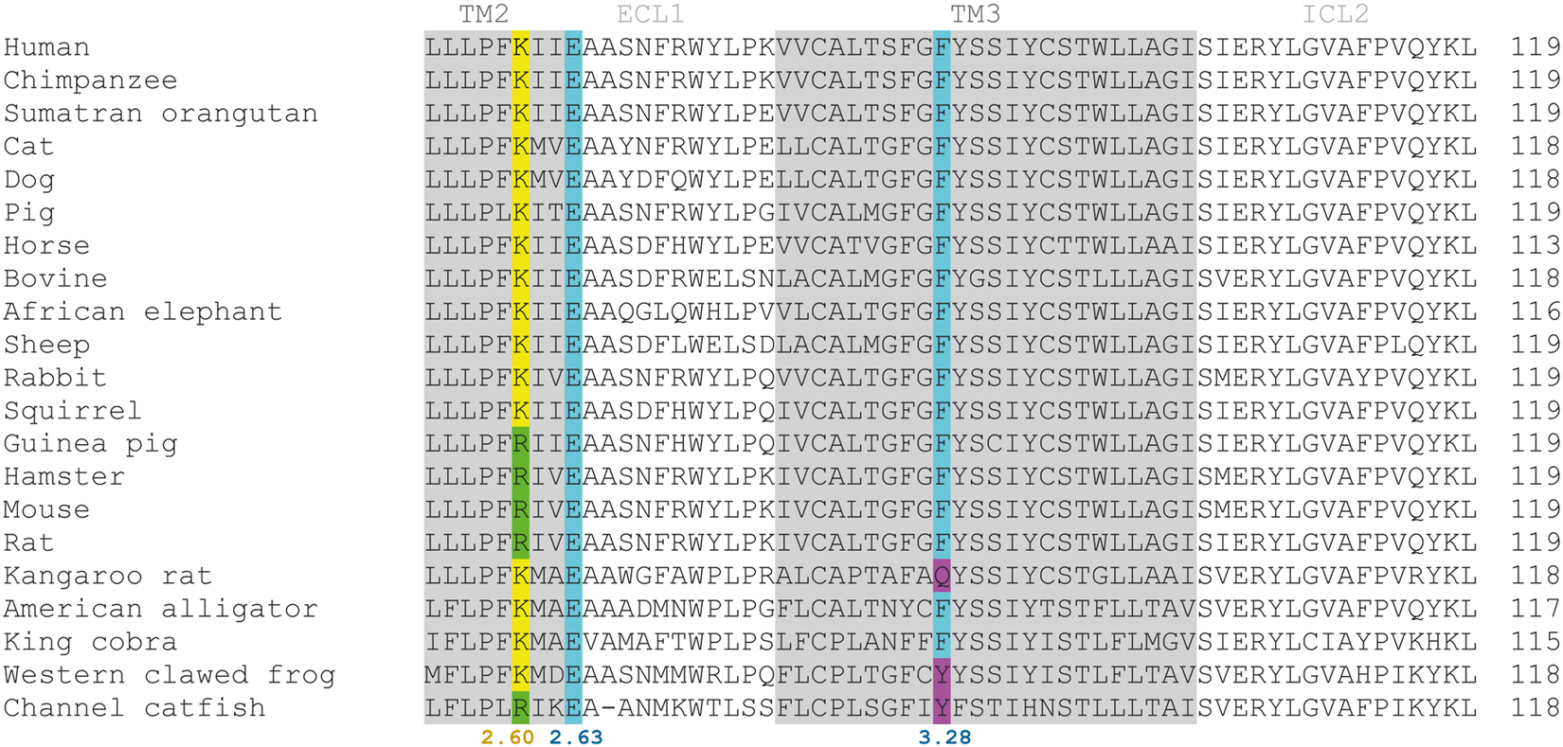
Sequence alignment of FFA2 orthologs. Clustal Omega alignments of the primary amino acid sequence of available orthologs of FFA2 using human residues 60 to 119 as reference. Whether Lys or Arg is present as residue 65 (location 2.60) is shown in color. Glu^68^ (location 2.63) is fully conserved and Phe^89^ (location 3.28) is also entirely conserved apart from in kangaroo rat, western clawed frog and channel catfish.

**Figure 8:**
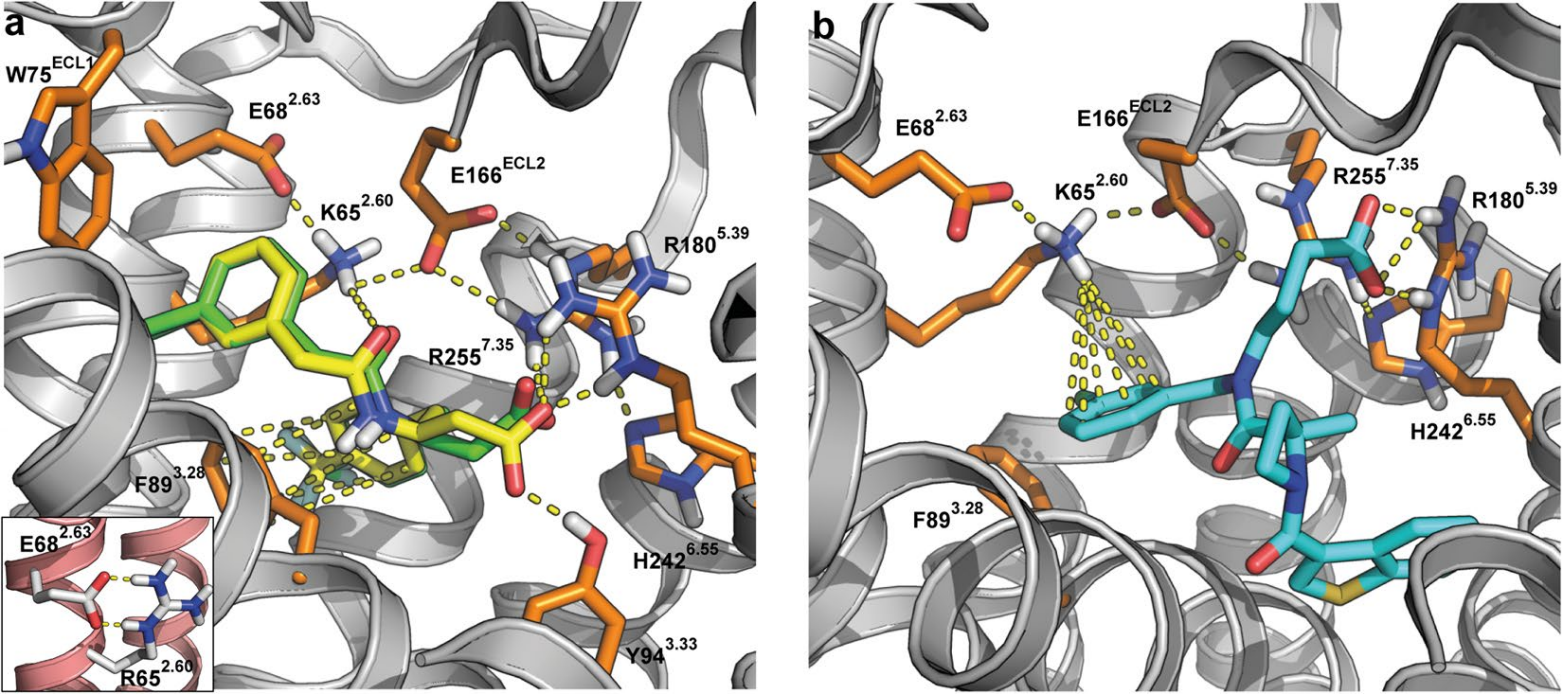
Predicted mode of binding of antagonists to Arg^65^Lys mouse FFA2. Docking of CATPB (**a**) and GLG0974 (**b**) into a homology model of mouse FFA2 containing an Arg^65^Lys alteration. (**a)** Docking position of CATPB to human FFA2 (*green*) is overlaid with the low energy pose obtained for CATPB in Arg^65^Lys mouse FFA2 (*yellow*). Insert to **A** illustrates that in the model of wild type mouse FFA2 the position of Arg^65^ is fixed via an ionic interaction with Glu^68^ (residue 2.63). (**b**) Illustration of binding of GLPG0974 to Arg^65^Lys mouse FFA2 and the importance of Lys at position 65.

To consider why CATPB and GLPG0974 and related molecules do not act as effective antagonists at mouse FFA2, we also compared sequence alignments. Although Phe^89^ and residues in close proximity within the primary sequence of human and mouse FFA2 are fully conserved (Fig. 7), the Lys^65^ residue in human FFA2 is replaced by Arg in mouse (Fig. 7). We were unable to obtain docking poses in which interactions with Arg^65^ were detected for either CATPB or GLPG0974 using a homology model of mFFA2. However, *in silico* substitution of Lys for Arg^65^ in this model resulted in a pose for CATPB that was indistinguishable from those obtained with the hFFA2 homology model (Fig. 8a). Whilst docking poses for GLPG0974 using Lys^65^Arg mFFA2 were distinct from those using wild type hFFA2 (Fig. 8b), GLPG0974 did, however, display important interactions with both Lys^65^ and Arg^180^ in this model (Fig. 8b). This may be why in studies using [^3^H]GLPG0974, although we observed each of high affinity binding of this ligand to wild type hFFA2, that such high affinity binding was eliminated by replacement of Lys^65^ by Arg and high affinity binding of [^3^H]GLPG0974 to wild type mFFA2 was lacking. Binding affinity generated by the reverse alteration, in which the Arg found in this position in mFFA2 was replaced by Lys, was some 7 fold lower than to wild type hFFA2.

To consider broader implications and to predict whether GLPG0974 would bind with high affinity to FFA2 orthologs from other species we searched more widely across available sequence data. This indicated that each of rat, hamster and guinea-pig FFA2 also has Arg at position 65 and, therefore, would not be predicted to bind GLPG0974 with significant affinity (Fig. 7). This variation seems to be largely restricted to rodents. One rodent that does not follow this pattern is kangaroo rat, which has Lys at this position and, as such, we predict that GLPG0974 would have high affinity at FFA2 in this species, although the replacement of Phe^89^ by Gln may confound this prediction. Moreover, from the modelling/ligand docking and functional studies we predict that antagonists related to CATPB will also show the same pattern of species selectivity. Given this analysis, it is clear why^14^ and^13^ were unable to explore the potential effectiveness of GLPG0974 in rodent models of disease and were forced to move directly to first-in-man clinical trials using this molecule without doing so. Clearly, a potential means to overcome this would be to generate lines of ‘humanized’ knock-in mice in which either the coding sequence of FFA2 was altered to the human form, or a single Arg^65^Lys alteration was introduced into the mFFA2 sequence. It is likely, however, that considering only Lys versus Arg identity at residue 65 in FFA2 as a predictor of high binding affinity for CATPB, GLPG0974 and related ligands will be too simplistic to explain this variation more broadly between species. For example, bovine FFA2 has Lys at residue 65 but does not bind CAPTB with high affinity (Hudson *et al*., unpublished). However, the loss and gain of function when switching these amino acids between primate and rodent orthologs of FFA2 is sufficient to provide important new insights into antagonist pharmacology at this receptor and may allow the synthesis of antagonists that have high affinity at wild type rodent orthologs of FFA2.

Interestingly, this is not the only study in which a Lys^65^ Arg mutation of hFFA2 has been employed. As confirmed herein Grundmann *et al*.^34^ showed that the agonist function of C3 was unaffected by this mutation, which will be discussed in more detail below. However, unlike the current studies in which both of the human specific antagonists GLPG0974 and CATPB lost substantial affinity at Lys^65^Arg hFFA2, Grundmann *et al*.^34^ reported CATPB to be an effective and competitive antagonist of C3 at this mutant. Although GLPG0974 was not assessed by Grundmann *et al*.^34^, their observations on the effectiveness of CATPB at this mutant are not compatible with either the current results, nor the inability of this compound to block rodent orthologs of FFA2, a feature that is well established^8,16^. Moreover, data shown here demonstrates across a range of assays that the ‘humanising’ Arg^65^Lys alteration in mFFA2 results in a gain of antagonist function, which confirms that antagonist species selectivity is indeed defined by the identity of the residue in position 65.

Sequence variation and alteration in ligand function between species orthologs of FFA2 is not restricted to antagonist pharmacology. In initial studies on Lys^65^Arg hFFA2 only modest variation was observed for the potency of either the endogenous agonist C3 or the synthetic FFA2 agonist compound 1. This is consistent with earlier studies that indicated, across a range of assays, whilst C3 is more potent at hFFA2 than at mFFA2, this difference is only in the region of three-fold^22,23^. Notably in this regard, Lys^65^Arg hFFA2 also showed some 2–3 fold reduction in potency for C3 compared to wild type hFFA2. The synthetic agonist compound 1 has also been described as being slightly more potent at hFFA2 compared to either mFFA2 or the rat ortholog^23^ and, again, such reduction in potency was also observed at Lys^65^Arg hFFA2 compared to the wild type receptor. More extensive effects on both agonists were observed, however, when the positive charge at residue 65 was either eliminated or converted to a negative charge. However, the degree of effect was markedly dependent upon the assay employed and which G protein-mediated signalling pathway was being assessed.

Although GPCRs are often classified as G_s_, G_i_ or G_q_-coupled, based on the predominant family of hetero-trimeric G proteins they interact with and activate to transduce intracellular signalling, this is routinely an oversimplification. This reflects that many receptors are appreciated to have the ability, as least when expressed in heterologous systems, to couple to members of more than one of the four broad subgroups of mammalian G proteins. Moreover, because the G_s_, G_i_ or G_q_-coupling preference of individual receptors is based most often on outcomes from assays that measure changes in amounts of intracellular 2^nd^ messengers, such classifications routinely ignore contributions of the G_12/13_-family G proteins as these interactions are more challenging to measure because they do not directly modulate 2^nd^ messenger levels. Contributions of G_s_, G_i_ or G_q_-family G proteins to the totality of canonical, G protein-mediated signalling can also be mapped via the use of bacterial toxins that modify various G protein α subunits^31^ and, more recently, small molecule inhibitors of the G_q/11_ group of G proteins^26^. However, only with the recent development of lines of HEK293 cells that have been genome-edited using CRISPR/Cas 9 technology^28–30^ has it been possible to explore the effect of re-introduction of individual G protein α subunits in engineered G protein-null backgrounds. Initial studies highlighted that although removal of positive charge from residue 65 of hFFA2 had, at most, modest effects on agonist potency at signalling endpoints that reflected activation of pertussis toxin-sensitive G_i_-family G proteins, in inositol monophosphate accumulation assays that reflect activation of G_q/11_ G proteins, much larger effects were recorded. This immediately suggested that elimination of positive charge at this residue generated a ‘biased’ form of the receptor at which signalling via G_i_ was preferred over signalling to G_q/11_ or to G_12/13_. In recent times ligand or receptor signalling ‘bias’ has frequently been taken to reflect differences in the ability of distinct ligands to harness interactions with a G protein compared to a non-canonical receptor interacting protein, most frequently an arrestin^18^. However, ligands that result in distinct patterns of receptor engagement with G protein subtypes compared to those produced by the (a) endogenous agonist clearly also ‘bias’ function of the receptor and this may have physiological consequences at least as important as altering selection between G protein and non-G protein-dependent signalling. Recent informatics studies have tried to understand and predict G protein selection by receptors^35,36^.

To potentially confirm and extend this hypothesis we employed a TGFα shedding assay that reports on activation of combinations of G_q/11_ and G_12/13_ family G proteins to probe the G protein selection profile of FFA2. As well as confirming the ability of orthosteric agonists to engage with members of each of the G_i_, G_q/11_ and G_12/13_ G protein groups these assays provided confirmation of the ‘biased’ nature of forms of hFFA2 lacking a positive charge at residue 65. Calculation of the extent of bias to favour signalling via G_i_ at these receptor variants was similar whether positive charge was simply removed or replaced with fixed negative change and was most extensive, in the region of 100 fold, for the endogenous agonist C3. This has considerable implications for function. Sequence alignments failed to identify any species in which a positively charged residue, either Lys or Arg, is not located at this position. However, once again it would be possible to generate transgenic knock-in mice in which G_q/11_-mediated signalling is predicted to be severely compromised compared to G_i_-mediated signalling. As we have shown previously^21^ although the role of FFA2 as an anti-lipolytic regulator in white adipocytes is transduced via G_i_, FFA2-mediated control of GLP-1 release from enteroendocrine cells is a G_q/11_-mediated process. As such, in mice expressing Arg^65^Ala or Arg^65^Glu mFFA2 (or indeed Lys^65^Ala or Lys^65^Glu hFFA2) we would predict that whilst C3-mediated regulation of lipolysis would be unaffected, SCFA-mediated regulation of GLP-1 release would be all but attenuated. Future studies will assess these hypotheses.

## Methods

### Materials

FFA2 ligands compound 1^23^ and AZ1729^21^ were synthesized as described previously. [^3^H]GLPG0974 (129 MBq/mL) was a gift of AstraZeneca (Molndal, Sweden). [^35^S]GTPγS was from PerkinElmer Life Sciences. Tissue culture reagents were from Invitrogen and molecular biology enzymes and reagents from New England BioLabs. All other experimental reagents were from Sigma-Aldrich unless indicated otherwise.

### Plasmids and mutagenesis

Human wild type and mutant FFA2 receptors with NanoLuc Luciferase fused to their N terminus were cloned into the pcDNA5/FRT/TO expression vector as described previously^37^ and used to generate doxycyline-inducible Flp-In T-REx HEK293 cell lines. For assays based on transient transfection a pcDNA5/FRT/TO vector expressing receptors fused to an HA-tag at their C terminus was cloned as previously described^38^. Site-directed mutagenesis to generate the point mutants of FFA2 was performed according to the QuikChange method (Stratagene, Cheshire, UK). The identity of all constructs was verified by nucleotide sequencing.

### Cell culture, transfection and generation of cell lines

HEK293 cells were used for experiments employing transient heterologous expression of receptors of interest. These cells were maintained in Dulbecco’s Modification of Eagle’s Medium (DMEM) supplemented with 10% fetal bovine serum, 2 mM L-glutamine and 1 x penicillin/streptomycin mixture (Sigma-Aldrich) at 37 °C with 5% CO_2_. Transfections were performed using polyethyleneimine and experiments carried out 48 h post-transfection. In experiments utilizing HEK293 cells with doxycycline-inducible stable receptor expression, the Flp-In T-REx system (Invitrogen) was used for cell line generation as described previously^39^. Medium for Flp-In T-REx HEK293 cells was additionally supplemented with 5 μg/ml blasticidin and 200 μg/ml hygromycin B. All experiments carried out using these cells were conducted after a 24 h treatment with 100 ng/ml doxycycline to induce expression of the receptor construct of interest.

### HTRF-based cAMP inhibition and inositol monophosphate accumulation assays

All cAMP and inositol monophosphate experiments were performed using Flp-In T-REx HEK293 cells induced to express the receptor of interest. Experiments were carried out using respective homogenous time-resolved FRET-based detection kits (CisBio Bioassays; CisBio, Codolet, France) according to the manufacturer’s protocol. For the cAMP inhibition assay cells were plated at 2000 cells/well in low-volume 384-well plates. The ability of agonists to inhibit 1 μM forskolin-induced cAMP production was assessed following a co-incubation for 30 min with agonist compounds. For the inositol monophosphate accumulation assay cells were plated at 7500 cells/well in low-volume 384-well plates and incubated for 2 h at 37 °C with test compounds. To assess ability of antagonists to inhibit agonist responses, cells were pre-incubated with antagonist compounds for 30 min at 37 °C prior to addition of an EC_80_ concentration of agonist. Respective reactions were stopped according to the manufacturer’s instructions and the output was measured by with a PHERAstar FS plate reader (BMGLabtech, Aylesbury, UK).

### Membrane preparation

Membranes were generated from Flp-In T-REx HEK293 cells treated with 100 ng/mL doxycycline to induce expression of receptor of interest. Cells were washed with ice-cold phosphate-buffered saline, removed from dishes by scraping and centrifuged at 3000 rpm for 5 min at 4 °C. Pellets were resuspended in TE buffer (75 mM Tris-HCl, 5 mM EDTA; pH 7.5) containing a protease inhibitor mixture (Roche Applied Science, West Sussex, UK) and homogenized with a 5 ml hand-held homogenizer. This material was centrifuged at 1500 rpm for 5 min at 4 °C and the supernatant was further centrifuged at 50000 rpm for 30 min at 4 °C. The resulting pellet was resuspended in TE buffer and protein content was assessed using a BCA protein assay kit (Pierce, Fisher Scientific, Loughborough, UK).

### [^35^S]GTPγS incorporation assay

Initially, 5 μg of generated membrane protein was pre-incubated for 15 min at 25 °C in assay buffer (50 mM Tris-HCl, 10 mM MgCl_2_; 100 mM NaCl; 1 mM EDTA; 1 μM GDP; 0.1% fatty acid-free bovine serum albumin; pH 7.4) containing the indicated ligand concentrations. The reaction was then initiated with addition of [^35^S]GTPγS at 50 nCi per tube, and the reaction was terminated after 1 h incubation at 25 °C by rapid filtration through GF/C glass filters using a 24-well Brandel cell harvester (Alpha Biotech, Glasgow, UK). Unbound radioligand was removed from filters by three washes with ice-cold wash buffer (50 mM Tris-HCl, 10 mM MgCl_2_; pH 7.4) and filters were dried for 2–3 h at room temperature. Dried filters were added to 3 mL of Ultima Gold^TM^ XR (PerkinElmer Life Sciences, Beaconsfield, UK) and [^35^S]GTPγS binding was determined by liquid scintillation spectrometry.

### Radioligand binding assay

Assays were carried out either with increasing concentrations (for saturation binding) or respective K_d_ concentrations (for displacement assays) of [^3^H]GLPG0974^15^, binding buffer (50 mM Tris-HCl, 100 mM NaCl, 10 mM MgCl_2_, 1 mM EDTA; pH 7.4), and the indicated concentrations of test compounds (for displacement assays) in a total assay volume of 200 µl in glass tubes. Binding was initiated by the addition of membranes (5 µg of protein per tube). All assays were performed at 25 °C for 2 h before termination by the addition of ice-cold phosphate-buffered saline and vacuum filtration through GF/C glass filters using a 24-well Brandel cell harvester (Alpha Biotech, Glasgow, UK). Each reaction well was washed three times with 2 ml of binding buffer. The filters were allowed to dry for 2–3 h and then placed in 3 ml of Ultima Gold^TM^ XR. Radioactivity was quantified by liquid scintillation spectrometry. Specific binding was defined as the difference between binding detected in the presence and absence of 10 µM unlabeled GLPG0974.

### TGFα shedding assay

A mixture of 250 ng AP-TGFα, 100 ng receptor of interest and 50 ng Gα protein were transfected using polyethyleneimine into one well of HEK293 cells cultured in a 12-well plate. Transfected cells were detached with PBS containing 0.05% trypsin and 0.52 mM EDTA and harvested by centrifugation at 190 × g for 5 min. The cell pellet was resuspended in PBS, followed by an incubation for 15 min at room temperature. After a second centrifugation at 190 × g for 5 min, pelleted cells were resuspended in 4 ml Hank’s Balanced Salt Solution (HBSS) containing 5 mM HEPES (pH7.4) per well in a 12-well plate. The cell suspensions were plated at 90 μl per well in a 96-well plate and incubated at 37 °C with 5% CO_2_ for 30 minutes. After the equilibration period 10 μl per well of 10× concentration of compounds were added and incubated for 1 h at 37 °C with 5% CO_2_. Plates were centrifuged at 190 × g for 2 min and 80 μL of supernatant was transferred into another 96-well plate. Solution containing p-NPP (10 mM p-NPP, 40 mM Tris-HCl (pH 9.5), 40 mM NaCl, 10 mM MgCl_2_) was added at 80 μl per well into supernatant-only plates as well as cell plates. Absorbance at 405 nm of both plates was read before and after a 2 h incubation at room temperature using a microplate reader (VersaMax, Molecular Devices). Detailed assay development and analysis has been described previously^27^.

### Homology modeling

Homology models of FFA2 receptors were constructed using the hFFA1 receptor (Protein Data Bank code 4PHU) as template^32^. The template was prepared for homology modeling as described previously^15^. The total sequence identity between FFA1 and FFA2 was determined to be 26%. The sequence identity between the transmembrane domains and hFFA2 and hFFA1 is 32%^40^. Key anchoring residue reported in this study (Lys^65^) and previously (Arg^180^, His^242^, Arg^255^)^15^ are all situated within the transmembrane domains of FFA2. Models of hFFA2, mFFA2, and Arg^65^Lys mFFA2 were generated using Prime’s homology modeling module (Prime, version 3.3, Schrödinger, LLC, New York). For mFFA2, Arg^65^ and Glu^68^ were manually paired up in an ionic lock, and all final models underwent restrained minimization using OPLS-2005 force field in Protein Preparation Wizard^41^.

### Ligand preparation and induced fit docking

Ligands (GLPG0974 and CATPB) were primed for docking using the OPLS-2005 force field in LigPrep (LigPrep, version 2.7, Schrödinger, LLC); ionization states were computed using Epik at pH 7.0 ± 2.0 (Epik, version 2.5, Schrödinger, LLC). Induced fit docking was executed for both antagonists using the IFD 2006 protocol (Glide version 5.9, Schrödinger, LLC; Prime version 3.2, Schrödinger, LLC). For hFFA2 the centroid of the binding site, into which GLPG0974 and CATPB were docked, was defined by residues: 14, 58, 61–69, 89, 90, 165, 166, 176, 179–181, 184, 242. Within hFFA2, residues ≤3 Å from each docked ligand were used to define the centroid of binding sites for mFFA2 and mFFA2-R65K. For all cases, trimming was executed for residues 89, 90, 165, 87, 145, 141, 179, while residues 65 and 255 were not refined during induced fit docking. Sampling of ligand conformations was performed using default settings; docking of ligands was executed using Glide Extra Precision (XP)^42^; a maximum number of 20 poses per ligand was allowed, and re-docking was executed in Glide XP mode for structures within 30 kcal/mol of the lowest energy protein-ligand complex. Residues refinement was set to ≤5 Å of each ligand pose. Poses were selected based on three criteria. First, and due to the key role of Lys^65^ in ligand binding and ortholog specificity, only docked ligands that actively engaged Lys^65^ were analyzed. Second, as a reference, our previous study on FFA2 antagonist binding^15^ was used as a guideline to how the carboxylate of docked ligands was oriented towards the key arginine residues (Arg^180^, Arg^255^). Third, to disregard unrealistic poses of compounds with high ligand strain, only the lowest energy poses fulfilling the aforementioned criteria were considered.

### Data analysis

All data are presented as means ± SEM of at least three independent experiments. Data analysis and curve fitting was carried out using the GraphPad Prism software package version 5.0b (GraphPad, San Diego). For functional assays the concentration-response data were plotted on a log axis, with the untreated vehicle control plotted at 1 log unit lower than the lowest ligand concentration, and fitted to a three parameter sigmoidal curve with the Hill slope constrained to equal 1. In case of inhibition experiments with antagonists an equivalent analysis was followed to fit an inverse sigmoidal curve. To perform the statistical analysis of curve parameters, data from multiple experiments were fitted independently and resulting curve fit values were analysed with indicated tests. For radioligand binding data, saturation binding curves were generated by fitting the specific binding, which was obtained by subtracting non-specific from total binding, to a one site specific binding model that allows calculation of K_d_ values for the radioligand at wild type and mutant receptors. To determine affinity of unlabelled ligands, data obtained in displacement assays were fit to an inverse three parameter sigmoidal curve constrained by radioligand affinity and concentration to allow for K_i_ calculation. To quantify the signaling bias that mutations of hFFA2 show compared to the wild type receptor, bias factor β was calculated by determining the logarithm of the ratio of relative intrinsic activities for a ligand at two different assays^43^. This approach requires only EC_50_ and E_max_ values of ligands in pathway 1 (cAMP or [^35^S]GTPγS assay) and 2 (TGFα shedding assay) at wild type and mutant receptors. Typically in such calculations a ligand of interest is assessed in comparison to a reference agonist that shows no bias between pathways 1 and 2, but in this case the response of agonist at wild type receptor serves as the reference to calculate the bias induced by respective mutations using the following equation.

$$\beta =\,\mathrm{log}({(\frac{{E}_{\max (P1)}}{E{C}_{50(P1)}}\frac{E{C}_{50(P2)}}{{E}_{\max (P2)}})}_{WT}\times {(\frac{{E}_{\max (P2)}}{E{C}_{50(P2)}}\frac{E{C}_{50(P1)}}{{E}_{\max (P1)}})}_{Mu\tan t})$$

### Data availability

The datasets generated and analysed during the current study are available from the corresponding author on reasonable request.
